# Dissecting the Serotonergic Food Signal Stimulating Sensory-Mediated Aversive Behavior in *C. elegans*

**DOI:** 10.1371/journal.pone.0021897

**Published:** 2011-07-21

**Authors:** Gareth Harris, Amanda Korchnak, Philip Summers, Vera Hapiak, Wen Jing Law, Andrew M. Stein, Patricia Komuniecki, Richard Komuniecki

**Affiliations:** Department of Biological Sciences, University of Toledo, Toledo, Ohio, United States of America; Duke University, United States of America

## Abstract

Nutritional state often modulates olfaction and in *Caenorhabditis elegans* food stimulates aversive responses mediated by the nociceptive ASH sensory neurons. In the present study, we have characterized the role of key serotonergic neurons that differentially modulate aversive behavior in response to changing nutritional status. The serotonergic NSM and ADF neurons play antagonistic roles in food stimulation. NSM 5-HT activates SER-5 on the ASHs and SER-1 on the RIA interneurons and stimulates aversive responses, suggesting that food-dependent serotonergic stimulation involves local changes in 5-HT levels mediated by extrasynaptic 5-HT receptors. In contrast, ADF 5-HT activates SER-1 on the octopaminergic RIC interneurons to inhibit food–stimulation, suggesting neuron-specific stimulatory and inhibitory roles for SER-1 signaling. Both the NSMs and ADFs express INS-1, an insulin-like peptide, that appears to cell autonomously inhibit serotonergic signaling. Food also modulates directional decisions after reversal is complete, through the same serotonergic neurons and receptors involved in the initiation of reversal, and the decision to continue forward or change direction after reversal is dictated entirely by nutritional state. These results highlight the complexity of the “food signal” and serotonergic signaling in the modulation of sensory-mediated aversive behaviors.

## Introduction

Olfactory acuity is modulated by nutritional status, with behaviors initiated by attractive stimuli generally enhanced by starvation and those initiated by repulsive (noxious) stimuli enhanced by satiety. Both monoamines, such as serotonin (5-HT), and neuropeptides translate nutritional status into olfactory modulation. Serotonergic signaling increases odor-evoked responses in moths and reduces sensory input and odor processing in mice, apparently coordinating sensory gain in the olfactory bulb with behavioral state [1], [2]. Similarly, insulin and leptin differentially modulate spontaneous and odor-evoked activity in rat olfactory neurons [3]. Serotonergic neurons often co-release classical neurotransmitters, such as glutamate, and neuropeptides, but our understanding of the nutritional modulation of serotonergic/peptidergic interactions is limited, given the complexity of the mammalian nervous system [4], [5]. To better understand serotonergic modulation of olfactory signaling, we have examined the food-dependent modulation of sensory-mediated locomotory behaviors in the model, *Caenorhabditis elegans*. In *C. elegans*, food dramatically alters monoaminergic signaling and modulates nutritionally-dependent locomotory transitions [6], [7]. Recent work suggests that 5-HT provides a balance of both excitatory and inhibitory input into key behaviors, through five different 5-HT receptors, most with orthologues in mammals [8], [9].

In *C. elegans*, food increases aversive responses mediated by the ASH sensory neurons through three 5-HT receptors, operating within the ASH-mediated locomotory circuit, but the relationship between food availability and serotonergic transmission or the source of the 5-HT activating these receptors has not been identified [10]–[12]. *C. elegans* contains at least nine serotonergic neurons; two NSMs, ADFs and HSNs that synthesize 5-HT directly and two AIMs and the RIH that do not express *tph-1*, required for serotonin synthesis, and instead accumulate secreted 5-HT through a 5-HT transporter, MOD-5 [13]. Interactions between NSM and ADF signaling are complex, with both neuron-specific and cooperative interactions described, although few studies have functionally localized the 5-HT receptors involved in NSM and/or ADF-dependent modulation [14]–[18].

In the present study, we have dissected the food signal that modulates ASH-mediated aversive behaviors and demonstrated that 5-HT released from the NSMs and ADFs functions antagonistically, with NSM 5-HT stimulating aversive responses and ADF 5-HT inhibiting food-stimulation. 5-HT from the NSMs and ADFs activates distinct subsets of 5-HT receptors, suggesting that food-dependent serotonergic signaling is characterized by changes in local 5-HT levels, involving primarily extrasynaptic 5-HT receptors. Finally, *ins-1* appears to act cell autonomously in both the NSMs and ADFs to inhibit serotonergic signaling. Together, these results highlight the complexity of serotonergic modulation and the obligate interactions among the multiple ligands released from the serotonergic neurons in the food-associated modulation of aversive behavior.

## Materials and Methods

### Materials

All reagents were purchased from Sigma Aldrich (St. Louis, MO). Neurochemicals were purchased from Sigma Aldrich (St. Louis, MO), restriction enzymes from New England Biolabs (Beverly, MA) and Promega (Madison, WI) and oligonucleotide primers from Integrated DNA Technologies (Coralville, IA). A *C. elegans* cDNA pool was purchased from OriGene Technologies (Rockville, MD), and additional cDNA pools were constructed from mixed stage mRNA using standard techniques. Green fluorescent protein (GFP) expression vectors were obtained from Andy Fire (Stanford School of Medicine).

### Cultures and maintenance of strains

The N2 Bristol WT isolate of *C. elegans* was used for all studies. All animals were raised at 20°C under uncrowded conditions [19]. The following mutant alleles were used in this study: *ins-1(tm1888)IV, mod-5(n822)I*, *ser-5(tm2647)I*, *ser-5(tm2654)I*, *ser-4(ok512)III*, *mod-1(ok103)V*, *ser-7(tm1325)X*, and *ser-1(ok345)X*. All strains were obtained from the *Caenorhabditis* Genetics Center (University of Minnesota, Minneapolis, MN) except, *ser-7(tm1325)X*, *ser-5(tm2647)I*, *ser-5(tm2654)I*, which were obtained from the National Bio-Resources Project (Tokyo Women's Medical University, Tokyo, Japan). Animals containing combinations of *null* or *gf* alleles were constructed using standard genetic techniques and confirmed by PCR. All mutant animals were backcrossed with the N2 Bristol strain at least 5× before use.

### Behavioral assays

Assay plates (5 cm NGM plates) were prepared daily and serotonin (4 mM) was added to NGM liquid media just prior to pouring. Dilute 1-octanol was prepared daily using 100% ethanol (vol/vol) [10], [20]. Synchronized fourth-stage larvae (L4) were picked 24 hrs pre-assay and assays were performed at 23–25°C. All assays were performed with blinded samples to remove experimental bias. Octanol avoidance was measured, as described by [10]. Briefly, the blunt end of a hair (Loew-Cornell 9000 Kolinsky 8 paintbrush), was taped to a toothpick, dipped in 30% 1-octanol and placed in front of an animal exhibiting forward sinusoidal locomotion. Time to reverse was recorded and assays were terminated after 20 sec, to minimize spontaneous reversals, which are effected by food availability [10], [21]–[23]. For assays in the absence of food or 5-HT, well-fed young adults (three to five per plate) were transferred to intermediate non-seeded plates and left for 1 min to prevent bacteria/media carry over, then transferred to NGM plates and assayed after 10 min. For assays in the presence of food (*E. coli* OP50) or 5-HT, animals were transferred to plates containing OP50 or 4 mM 5-HT and assayed after 20 and 30 min, respectively.

**Figure 1:**
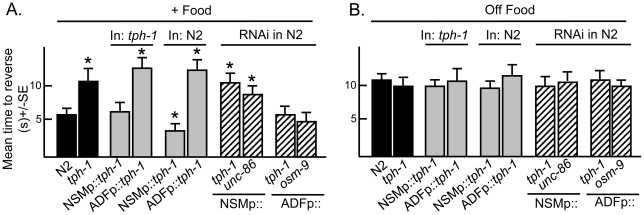
The serotonergic NSM and ADF neurons modulate the food-dependent stimulation of aversive responses by the ASH sensory neurons. Wild-type, mutant, transgenic and RNAi expressing animals were examined for aversive responses to dilute 1-octanol (30%) in the presence (A) or absence (B) of food, as described in Methods. NSM and ADF selective promoters: (*ceh-2*, I3, M4, NSM, M3) and (*srh-142*, ADF), respectively [**51]**, [52****]. Data are presented as a mean ± SE and analyzed by two-tailed Student's *t* test. “*” P<0.001, significantly different from wild-type animals incubated in the presence of food.

Post-initiation assays were performed as follows: synchronized fourth-stage larvae (L4) were picked 24 hrs pre-assay. Octanol avoidance was measured as above, except that animals were examined for duration of reversal by 1) counting the number of head swings per reversal and 3) determining the angle from the initial trajectory once reversal was complete (Figure 1). Twenty animals/strain/condition were assayed. Data are presented as a mean ± SE and analyzed by two-tailed Student's *t* test. *, P<0.001.

### Rescue constructs and RNAi

All rescue constructs were created by overlap fusion PCR or by cloning into pPD95.75 [24], [25]. The following neuron-selective promoters were used: *srh-142p* (ADF), *ceh-2p* (I3, M4, NSM, M3), *tdc-1p* (RIC, RIM, spermetheca). For overlap PCR, constructs were pooled from at least 3 reactions and were co-injected with *myo-3p::gfp*, *F25B3.3p::gfp* or *rol-6* and carrier DNA (to 100 ng) into gonads of wild-type and null mutant animals by standard techniques [24], [26]. At least three lines were examined and all constructs in pPD95.75 were confirmed by sequencing.

Neuron selective/specific RNAi transgenes were constructed as described by [27] by fusing neuron-selective promoters to exon rich regions of target genes. Exon-rich regions were amplified using forward and reverse primers to create template A from either cDNA or genomic DNA by PCR-fusion, as previously described by [25]. Neuron-selective promoters were amplified using a forward primer with reverse (sense) or reverse (antisense) primers to create templates B and C, respectively [27]. Templates A and B were fused using a forward internal promoter primer and a reverse internal target gene primer to create the sense construct (Product D). Templates A and C were fused using the forward internal promoter primer and the forward internal target gene primer to create the antisense construct (Product E). At least three products were pooled and sense and antisense transgenes were microinjected at 25–100 ng/µl. At least 3 transgenic lines were examined for each RNAi. All strains are listed in Strain List S1. Primer sequences used for RNAi and rescue construct generation are shown in Table S1 and S2.

## Results

### Serotonergic signaling from the NSMs, but not the ADFs, is required for the food-stimulation of aversive responses mediated by the ASH sensory neurons

Food or 5-HT stimulate ASH-mediated aversive responses to dilute (30%) 1-octanol and *tph-1* null animals that lack tryptophan hydroxylase, the rate-limiting enzyme for 5-HT biosynthesis, do not increase aversive responses on food [10]–[12]. As noted above, *C. elegans* contains at least nine serotonergic neurons and 5-HT from the NSMs and ADFs, the major 5-HT synthesizing neurons surrounding the nerve ring, appear to function independently to modulate behavior [14], [15], [17]. Indeed, both the NSMs and the ADFs have been previously implicated in a number of serotonin-dependent, sensory-mediated, behaviors [14]–[18].

Therefore, to better understand the serotonergic “food signal,” in the modulation of ASH-mediated aversive responses, the individual roles of the major 5-HT synthesizing neurons in the nerve ring, the NSMs and ADFs, in food-stimulation were dissected, using neuron-specific rescue and RNAi. Since Esposito first described neuron-specific RNAi knockdown in individual pairs of sensory neurons in 2007, many labs, in addition to our own, have used this technique effectively [12], [27]–[30]. In fact, we have found that the major problem with this RNAi technique is not whether it effectively knocks down expression (it usually does), but whether the RNAi will spread to additional tissues, especially if it is more broadly expressed (i.e., muscle or motorneurons), significantly complicating the interpretation of any neuron-specific results (Komuniecki, unpublished). However, it is important to note that as this technique is more broadly applied, each neuron has the potential to respond differently to RNAi, emphasizing the need for independent corroboration of any RNAi result. In examining the roles of NSM and ADF signaling below, we have confirmed any neuron specific RNAi result by the neuron-specific rescue of null mutants, the RNAi knockdown of two different genes predicted to decrease serotonergic signaling, and most importantly, the neuron-specific RNAi knockdown of genes predicted to increase serotonergic signaling (mod-5) that yield phenotypes opposite to those observed for the inhibition of serotonergic signaling. In addition, to control for any potential RNAi spreading, we have also used NSM and ADF specific RNAi to knockdown the expression of ser-5, a receptor essential for food stimulation but not expressed in the NSMs or ADFs. As predicted, ser-5RNAi knockdown in the ASHs abolished food-stimulation, but ser-5RNAi knockdown in the NSMs or ADFs had no effect (Komuniecki, data not shown) [12].

The food-dependent stimulation of aversive responses in *tph-1* null animals was rescued by the expression of *tph-1* in the NSMs, but not the ADFs ((*ceh2p::tph-1(+)* vs *srh-142p::tph-1(+)*; Figure 1). Similarly, food-stimulation was abolished by the tph-1RNAi knockdown in the NSMs, but not in the ADFs, of wild-type animals (Figure 1). To confirm a role for the NSMs in food-stimulation, RNAi was also used to selectively knockdown *unc-86* in the NSMs and *osm-9* in the ADFs, on the observation that UNC-86 (POU domain transcription factor) and OSM-9 (TRPV channel subunit) were essential for 5-HT synthesis in the NSMs and ADFs, respectively [15], [31] (Figure 1). As predicted, the NSMp::unc-86RNAi abolished food-sensitization, but ADFp::osm-9RNAi had no effect on aversive responses (Figure 1). Thus, using three different approaches, we have demonstrated that that 5-HT signaling from the NSMs, but not the ADFs, is required for stimulation of ASH-mediated aversive responses by food. Conversely, as predicted none of these treatments has any effect off food (Figure 1B).

### Serotonergic signaling from the ADFs abolishes the stimulation of ASH-mediated aversive responses by food

**Figure 2:**
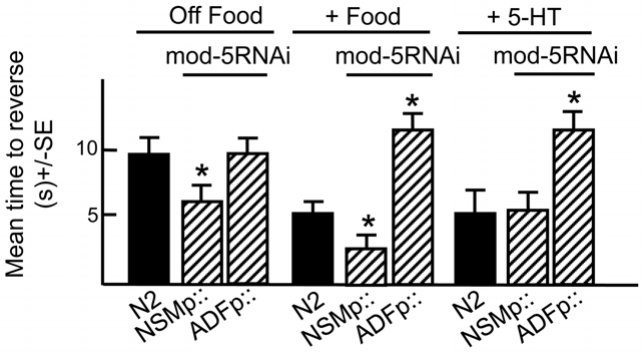
The neuron-selective increase in NSM or ADF serotonergic signaling by RNAi knockdown of *mod-5* that encodes a 5-HT reuptake transporter differentially modulates aversive responses. Wild-type, mutant, transgenic and RNAi expressing animals were examined for aversive responses to dilute 1-octanol (30%) in the presence or absence of food and/or 5-HT (4 mM), as described in Methods. Data are presented as a mean ± SE and analyzed by two-tailed Student's *t* test. “*” P<0.001, significantly different from wild-type animals incubated in the presence of food.

To further define the roles of serotonergic signaling from the NSMs and ADFs, *mod-5*, which encodes a 5-HT reuptake transporter, was selectively knocked down in the NSMs or ADFs, using RNAi [27], [32]. These studies are based on the assumption that the neuron-specific knockdown of mod-5 would selectively modulate signaling from these neurons, but more global effects on other 5-HT pools cannot be ruled out. Reassuringly, the RNAi knockdown of mod-5 in either the NSMs or ADFs had different effects on octanol sensitivity. As predicted, *mod-5* null animals and wild-type animals with *mod-5* knocked down in the NSMs exhibited increased aversive responses both off and on food when compared to wild-type animals, presumably driven by the accumulation of 5-HT released from the NSMs (Figure 2). Similarly, NSMp::*tph-1* overexpression in wild type animals also increased aversive responses on food (Figure 1A). Interestingly, NSMp::*tph-1* overexpression had no effect off food, presumably because food-stimulation of the NSMs was required for 5-HT release (Figure 1B). In contrast, ADFp::mod-5RNAi abolished food-stimulation, suggesting that 5-HT released from the ADFs inhibited food-stimulation, but not basal ASH signaling (data not shown, Figure 2). Indeed, ADFp::*tph-1* overexpression in wild-type animals also abolished food-stimulation (Figure 1A). These results confirm a stimulatory role for NSM 5-HT and suggest that ADF 5-HT inhibits the stimulation of ASH-mediated aversive responses on food.

### INS-1 from the NSMs and ADFs inhibits serotonergic signaling

**Figure 3:**
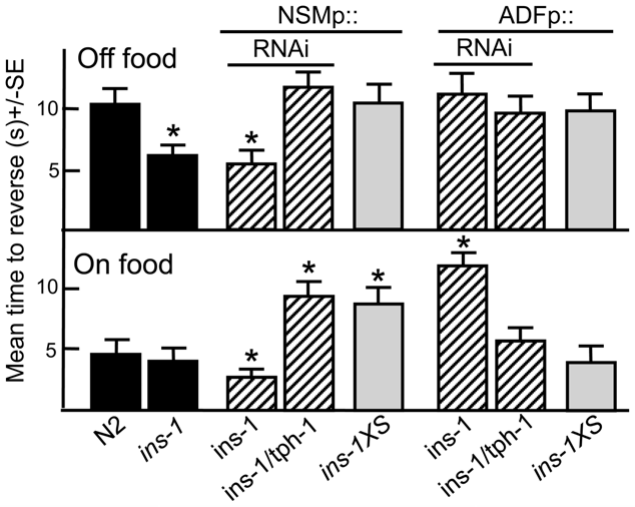
Insulin signaling modulates 5-HT release from the ADFs and NSMs. Wild-type, mutant, transgenic and RNAi expressing animals were examined for aversive responses to dilute 1-octanol (30%) in the presence or absence of food, as described in Methods. Data are presented as a mean ± SE and analyzed by two-tailed Student's *t* test. “*” P<0.001, significantly different from wild-type animals incubated in the presence or absence of food.

The insulin-like peptide, INS-1 is expressed in the both NSMs and ADFs and *ins-1* null animals exhibited more rapid aversive responses than wild-type animals off food, suggesting that INS-1 inhibited ASH-mediated aversive behavior [**33]**, [34****] (Figure 3). To determine if this *ins-1* phenotype was mediated by INS-1 from the NSMs and/or ADFs, *ins-1* was selectively knocked down using RNAi. NSMp::ins-1RNAi stimulated aversive responses both on and off food (Figure 3). In contrast, NSM *ins-1* overexpression in wild-type animals abolished food-dependent increases in aversive responses (Figure 3). Importantly, animals with both *tph-1* and *ins-1* knocked down in the NSMs did not exhibit the more rapid aversive responses off food observed after the NSM knockdown of *ins-1* alone, suggesting that NSM INS-1 inhibited serotonergic signaling from the NSMs (Figure 3). In contrast, ADFp::ins-1RNAi abolished food-stimulation, suggesting that *ins-1* knockdown in the ADFs might stimulate ADF serotonergic signaling and inhibit aversive responses, in agreement with observations described above for the ADF knockdown of *mod-5* or overexpression of *tph-1* (Figure 3). Indeed, animals with both *ins-1* and *tph-1* knocked down in the ADFs exhibited wild-type responses that were stimulated by food (Figure 3). These results suggest that ADF INS-1 may have an autocrine function in the ADFs and inhibits ADF serotonergic signaling. However, the possibility that INS-1 in acting in parallel to or downstream of serotonergic signaling cannot be ruled out.

### Distinct 5-HT receptors are involved in the NSM stimulation and ADF inhibition of aversive responses

**Figure 4:**
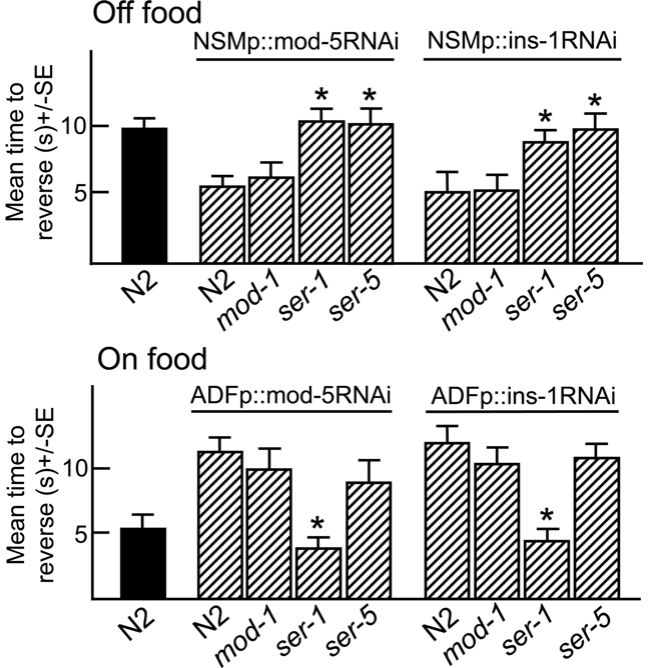
Serotonergic signaling from the NSMs or ADFs requires unique subsets of 5-HT receptors in the modulation of ASH-mediated aversive responses. Wild-type and 5-HT receptor null mutants expressing neuron selective *mod-5* or *ins-1* RNAi transgenes (Top: NSMp::RNAi, Bottom: ADFp::RNAi) were examined for aversive responses to dilute 1-octanol (30%) in the presence or absence of food, as described in Methods.

**Figure 5:**
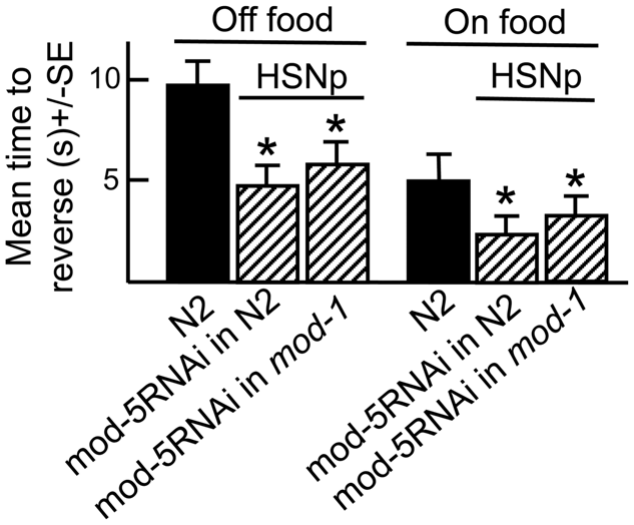
5-HT from the HSNs increases aversive responses on and off food. Wild-type and 5-HT receptor null animals expressing neuron selective mod-5RNAi were examined in the presence or absence of food for aversive responses to dilute 1-octanol (30%). Data are presented as a mean ± SE and analyzed by two-tailed Student's *t* test. “*” P<0.001, significantly different from wild-type animals incubated in the presence or absence of food.

As noted above, NSM 5-HT is essential for the stimulation of aversive responses by food, while ADF 5-HT abolishes food-stimulation, suggesting that food and/or ASH-mediated signaling may inhibit serotonergic signaling from the ADFs. To identify the specific 5-HT receptors mediating the effects of NSM or ADF 5-HT, aversive responses were examined in animals with null alleles for *ser-1*, *ser-5* or *mod-1*, the three 5-HT receptors previously identified as playing a role in 5-HT-dependent aversive responses, after RNAi knockdown of either *mod-5* or *ins-1* in the NSMs or ADFs, on the assumption that knockdown should specifically increase serotonergic signaling from either neuron pair [11]. NSMp::ins-1RNAi or NSMp::mod-5RNAi increased aversive responses both on and off food in both wild-type and *mod-1* animals, but had no effect on aversive responses in *ser-1* or *ser-5* animals, suggesting that NSM serotonergic signaling required both SER-1 and SER-5 (Figure 4, data not shown). MOD-1 is also essential for food or 5-HT-dependent stimulation, so that the ability of NSM 5-HT to stimulate aversive responses in *mod-1* null animals was surprising [11]. Interestingly, mod-5RNAi knockdown in the third pair of 5-HT biosynthetic neurons, the HSNs, using the *egl-47* promoter, also increased basal and 5-HT-stimulated aversive responses in both wild-type and *mod-1* null animals, suggesting either that MOD-1 is not required for 5-HT stimulation from any of the biosynthetic neurons or that the levels of 5-HT released after *mod-5* knockdown are sufficient to overcome the need for MOD-1 (Figure 5). In contrast, ADFp::ins-1RNAi or ADFp::mod-5RNAi inhibited food stimulation in wild-type, *ser-5*, and *mod-1* null animals, but had no effect in *ser-1* null animals, suggesting that the activation of SER-1 by ADF 5-HT inhibited food-stimulation (Figure 4). This result was also surprising, as previous work demonstrated that the expression of *ser-1* in the RIA interneurons, major downstream synaptic partners of the ADFs, was essential for food or 5-HT stimulation and suggested that ADF SER-1 signaling antagonized NSM SER-1 signaling in the RIAs [11].

**Figure 6:**
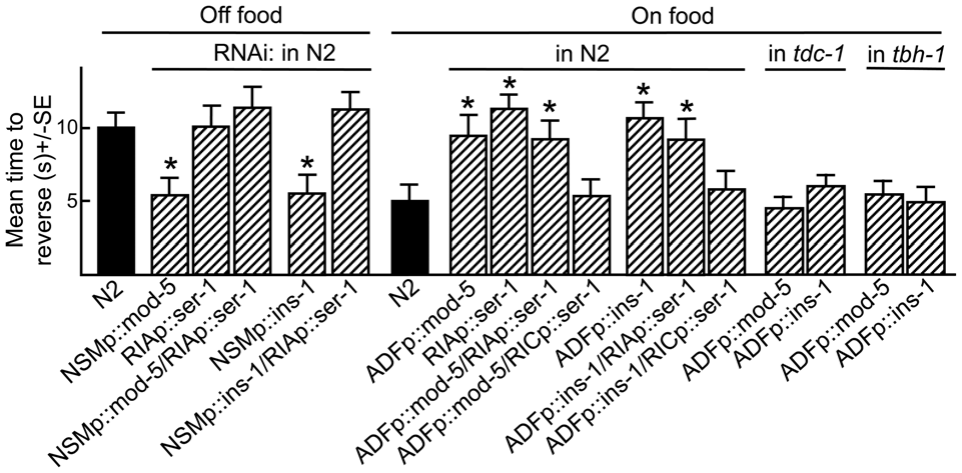
NSM 5-HT activates SER-1 in the RIA interneurons to stimulate aversive responses and ADF 5-HT activates SER-1 in the octopaminergic RIC interneurons to inhibit food-stimulation. Wild-type and transgenic animals were assayed for aversive responses to dilute 1-octanol (30%) in either the presence or absence of food. Neuron selective promoters for RIA and RIC neurons: (*glr-3*, RIA) and (*tdc-1*, RIC, RIM, spermetheca), respectively [**53]**, [54****]. Data are presented as a mean ± SE and analyzed by two-tailed Student's *t* test. “*” P<0.001, significantly different from animals incubated in the presence or absence of food.

To identify the neuron(s) involved in NSM and ADF SER-1 signaling, neuron-specific RNAi was used to create transgenic animals expressing different combinations of RIAp::ser-1, NSMp::ins-1, NSMp::mod-5, ADFp::ins-1, and ADFp::mod-5RNAi. The more rapid aversive responses observed after NSMp::ins-1RNAi or NSMp::mod-5RNAi were absent when *ser-1* was also knocked down in the RIAs, supporting our hypothesis that food-stimulation and increased serotonergic signaling from the NSMs required SER-1 in the RIAs (Figure 6) [11]. Importantly, NSMp::ins-1or NSMp::mod-5RNAi in *ser-1* mutant animals phenocopies their double RNAi, supporting the effectiveness of this double RNAi approach (compare Figures 4 and 6). In contrast, although the inhibition of food-stimulation observed after ADFp::ins-1 or ADFp::mod-5RNAi knockdown was absent in *ser-1* null animals, it was unaffected by the simultaneous knockdown of *ser-1* in the RIAs, suggesting that the ADF SER-1 inhibition of food-stimulation involved other neurons (Figure 6).

Interestingly, ADFp::mod-5RNAi or ADFp::ins-1RNAi did not inhibit food-stimulation in *tdc-1* or *tbh-1* null animals that lack octopaminergic signaling, suggesting that the ADF-dependent inhibition of food-stimulation might require OA (Figure 6). *ser-1p::gfp* is robustly expressed in both the RIAs and the octopaminergic RICs [22]. As predicted, aversive responses in animals with ADFp::mod-5/RICp::ser-1 or ADFp:ins-1/RICp::ser-1 knocked down by RNAi were still stimulated by food (Figure 6). Together, these results suggest that NSM 5-HT activates SER-1 in the RIAs to stimulate aversive responses and ADF 5-HT activates SER-1 in the RICs to stimulate OA release and inhibit food-stimulation.

### NSM 5-HT also modulates locomotory behavior after reversal is initiated

**Figure 7:**
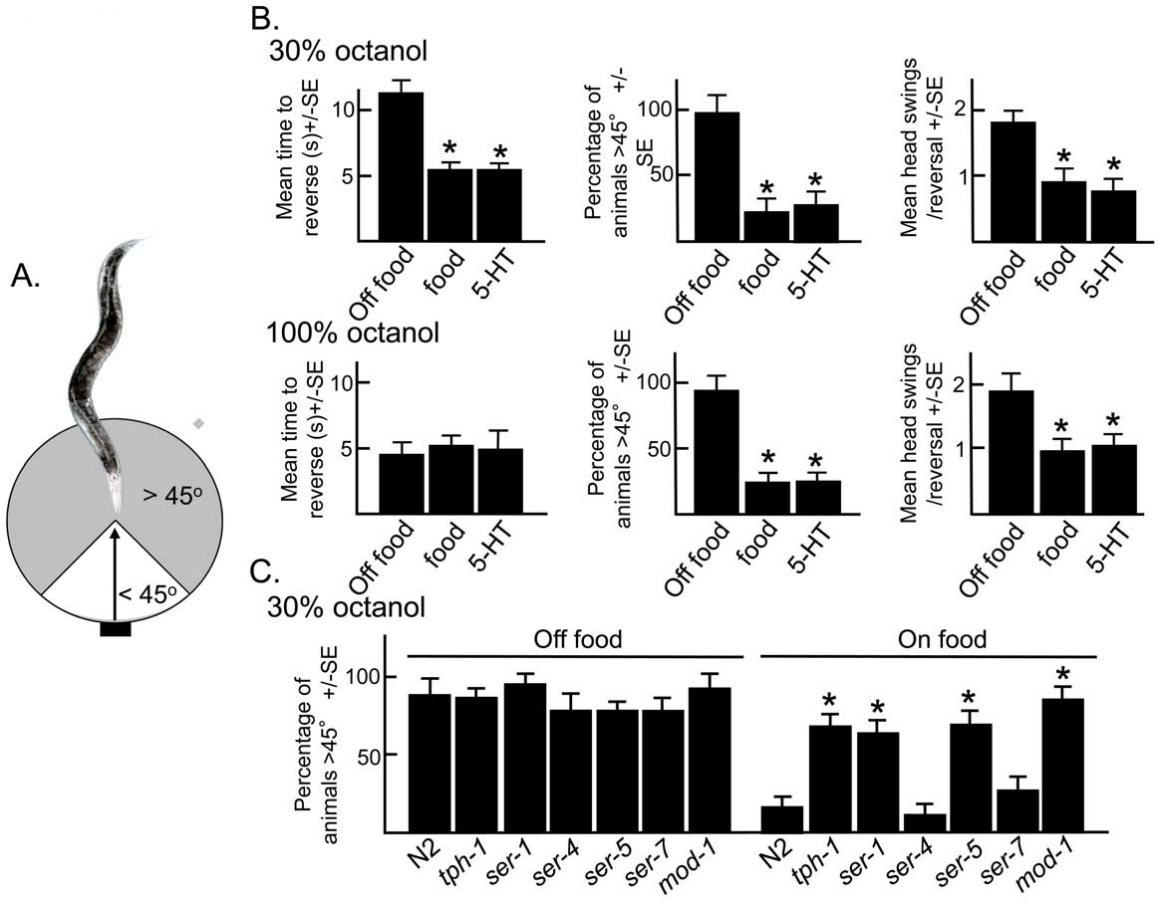
Food and 5-HT modulate locomotory behavior after reversal is initiated. **A.** Diagram outlining the measurement of post-initiation behaviors. **B.** Wild-type animals were examined for post-initiation responses in the presence or absence of food or 5-HT (4 mM), as described in Methods. After reversal was complete, the angle at which the animals resumed forward locomotion, relative to their initial trajectory, was recorded, i.e., the angle of animals proceeding along the same path was 0, and for animals proceeding in the opposite direction was 180. In addition, animals were examined for reversal duration (number of heads swings). **C.** Locomotory trajectory after the completion of reversal was assayed in mutant animals in the presence and absence of food. Data are presented as a mean ± SE and analyzed by two-tailed Student's *t* test. “*” P<0.001, significantly different from animals incubated in the presence or absence of food.

In addition to stimulating the initiation of reversal (time to reverse after exposure to stimulus), food also decreased reversal length (head swings/reversal) and directional decisions after reversal was complete (Figure 7A, B). For example, on food reversals were short (<1 head swing/reversal) and after reversal was complete most animals continued forward along their previous path (<45° from initial trajectory). In contrast, off food animals backed up more extensively and turned significantly away from their previous trajectory (>45° from initial trajectory). Surprisingly, these post-initiation phenotypes were independent of the intensity of the initiating stimulus, i.e., even though animals initiated reversal much more rapidly to 100% than 30% 1-octanol, food had identical effects on post-initiation responses, suggesting nutritional state and not intensity of the noxious stimulus dictated these aversive responses (Figure 7B). Exogenous 5-HT mimicked food in these post-initiation assays and modulated the length and directionality of reversal, and, as predicted, post-initiation responses in *tph-1* null animals on food were similar to those of animals off food (Figure 7). SER-1, SER-5 and MOD-1, the 5-HT receptors essential for food stimulation of aversive responses, were also essential in the food-dependent modulation of post-initiation responses (Figure 7C).

**Figure 8:**
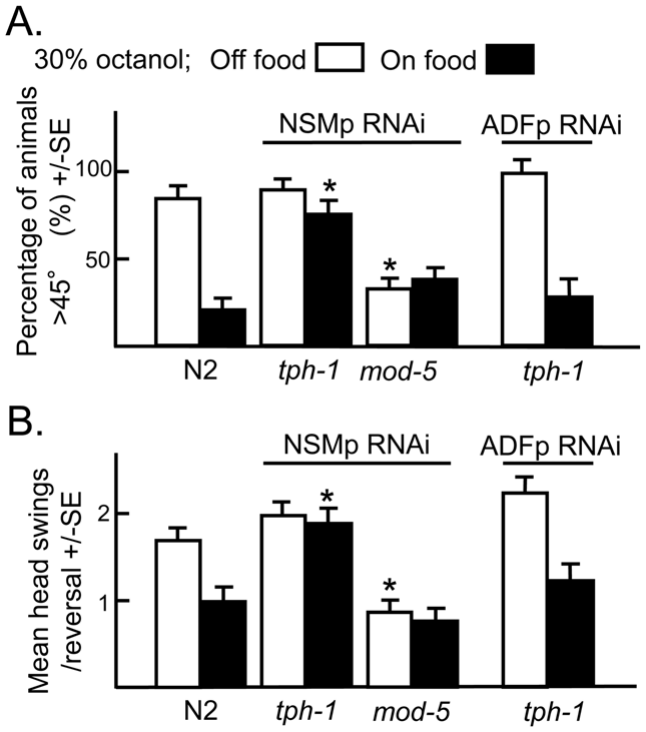
Food-dependent modulation of post-initiation behaviors requires NSM, but not ADF 5-HT. Wild-type animals and animals expressing neuron-selective RNAi were examined for post-initiation behaviors in the presence or absence of food. **A.** After reversal was complete and the angle at which the animals resumed forward locomotion, relative to their initial trajectory, was recorded, i.e., the angle of animals proceeding along the same path was 0, and for animals proceeding in the opposite direction was 180. **B.** Head swings per reversal, as described in Methods. Data are presented as a mean ± SE and analyzed by two-tailed Student's *t* test. “*” P<0.001, significantly different from wild-type animals incubated in the presence or absence of food.

To identify the source of 5-HT responsible for food-dependent modulation of post-initiation behaviors, animals with altered NSM and ADF signaling were examined on food (Figure 8). Food-dependent alterations in post-initiation behaviors were abolished by NSMp::tph-1RNAi, but were unaffected by ADFp::tph-1RNAi, suggesting 5-HT from the NSMs, but not the ADFs was required for modulation of post-initiation responses on food. As predicted, increasing NSM serotonergic signaling by NSMp::mod-5RNAi suppressed turns and reversal duration off food (Figure 8). These results suggest that NSM 5-HT not only increased responsiveness to ASH-mediated odorants, but also markedly altered post-initiation behaviors, regardless of the intensity of the initiating aversive stimulus. This result suggests that NSM signaling may provide differential input into potentially overlapping locomotory circuits to modulate both acute aversive responses and post-initiation locomotory behaviors.

## Discussion

**Figure 9:**
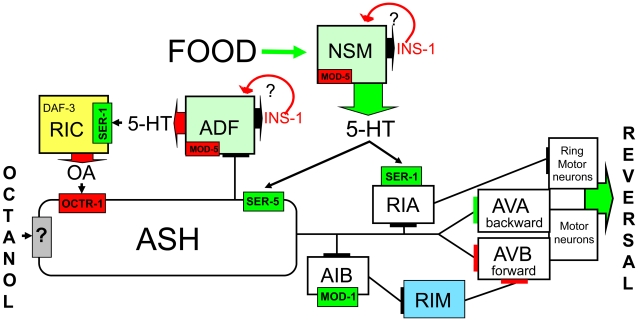
Model for the food dependent modulation of the ASH-mediated aversive behavior. On food, the release of 5-HT from the NSMs stimulates ASH-mediated aversive responses. In contrast, 5-HT from the ADFs and OA from the RICs inhibits 5-HT sensitization. ASH, sensory neuron necessary and sufficient for aversive responses to dilute octanol; AVA, backward command interneuron; AVB, forward command interneuron; INS-1, insulin-like peptide; OA, octopamine; OCTR-1, α-adrenergic-like OA receptor; SER-1, 5-HT_2_-like receptor; SER-5; 5-HT_6_-like receptor; MOD-1; 5-HT-gated chloride channel; Green (NSM and ADF serotonergic neurons expressing TPH-1); MOD-5, 5-HT reuptake transporter; Yellow (RIC octopaminergic neurons expressing TDC-1 and TBH-1); Blue (RIM tyraminergic neurons expressing TDC-1).

Food availability and nutritional status modulate olfactory acuity and behavior in most organisms [35]–[39]. In *C. elegans*, food stimulates ASH-mediated aversive responses and the present studies highlight the complexity of the serotonergic signal defining food availability (Figure 9). 5-HT released from the serotonergic NSMs and ADFs appears to have antagonistic effects on octanol-stimulated aversive behavior, with NSM 5-HT stimulating aversive responses and ADF 5-HT inhibiting food-stimulation. For example, increasing serotonergic signaling from the NSMs by knocking down NSM *mod-5* or *ins-1* or overexpressing *tph-1* stimulates basal aversive responses to levels observed on food or exogenous 5-HT. Conversely, increasing serotonergic signaling from the ADFs by knocking down ADF *mod-5* or *ins-1* or overexpressing *tph-1* abolishes the stimulation of aversive responses by food or exogenous 5-HT. These genetic manipulations have been interpreted as having acute effects on serotonergic signaling, but it is worth noting that altered serotonergic signaling can potentially also have effects on neuronal development in *C. elegans* [40]. These results highlight the complexity of serotonergic modulation and the potential limitations of exogenous ligands in characterizing serotonergic signaling.

Interactions between NSM and ADF signaling are complex, with both neuron-specific and cooperative interactions described previously. The branched NSMs make synapses onto the pharyngeal basement membrane and muscle, but also secrete 5-HT and neuropeptides directly into the pseudocoelomic fluid from vesicle-filled varicosities [13], [41]. Many mammalian monoaminergic neurons are also “asynaptic” [4]. In contrast, the ADFs are innervated by the ASHs directly and synapse onto interneurons modulating key locomotory transitions [42]. The NSMs are required for the enhanced slowing response and both the NSMs and ADFs are required for dispersal during starvation [16], [18]. However, these studies often have relied on NSM ablation, so whether NSM 5-HT, glutamate and/or neuropeptides were involved is unclear. In contrast, the food-sensitization of hyperoxia avoidance and aversive learning were rescued in *tph-1* animals by *tph-1* expression in the ADFs, but not the NSMs, supporting a role for ADF 5-HT in food-sensitization [14], [15]. Whether the ADFs sense food directly or respond to signals from other neurons in response to food availability/quality is unclear. However, ADFp::*tph-1* expression is elevated in *daf-7* animals [14]. *daf-7* encodes a TGF-β homologue and the ASI expression of DAF-7 decreases during stress/starvation, suggesting that starvation stimulates ADF 5-HT and food stimulates NSM 5-HT [14], [43], [44]. Serotonergic signaling from ADFs requires MOD-1 for avoidance to pathogenic bacteria [15]. Bacteria should stimulate NSM 5-HT and activate feeding, but pathogenic bacteria may also stimulate ADF 5-HT to antagonize NSM signaling, with products from the pathogens either activating the ADFs directly or indirectly through other sensory neurons. In the present studies, SER-1, not MOD-1, was essential for the ADF 5-HT inhibition of food-stimulation and it is unclear if SER-1 was required for aversive learning to pathogenic bacteria, highlighting the potential complexity of ADF serotonergic signaling.

Food and 5-HT stimulate ASH-mediated aversive responses through at least three different 5-HT receptors, operating within the ASH-mediated circuit [10], [11], [12]. 5-HT from the NSMs, ADFs and potentially other serotonergic neurons differentially interacts with subsets of these 5-HT receptors and serotonergic signaling appears to involve precisely modulated changes in local 5-HT levels, mediated primarily by extra-synaptic 5-HT receptors (Figure 9). In fact, *C. elegans* 5-HT receptors are expressed on many neurons that are not directly innervated by serotonergic neurons, suggesting that most serotonergic signaling is humoral and extra-synaptic [22], [32], [45]. Our data suggest that stimulation of aversive responses by NSM 5-HT requires SER-5 and SER-1, and confirm the role of the RIAs in NSM 5-HT stimulation [11]. In contrast, MOD-1 is not required, although it is essential for the sensitization of aversive responses by food or exogenous 5-HT, suggesting that additional sources of 5-HT may contribute to food-sensitization or that the levels of 5-HT released from the NSMs in these experiments overcome the need for MOD-1. The inhibition of food-stimulation by ADF 5-HT requires only SER-1, not in the RIAs but in the RICs. Presumably the Gα_q_-coupled SER-1 stimulates OA release from the RICs that, in turn, inhibits ASH signaling through the Gα_o_-coupled OCTR-1, as we have demonstrated previously [12], [46]. Together, these results demonstrate that SER-1 is required for both ADF inhibition and NSM stimulation, highlighting the complexity of serotonergic modulation.

Nutritional status modulates multiple aspects of the ASH-mediated aversive response, including reversal duration and trajectory after reversal is complete. For example, off food, animals initiate reversal more slowly, back up further and are more likely to turn from their initial trajectory, often initiating an omega turn. These post-initiation responses are independent of the intensity of the initiating stimulus (30 vs 100% 1-octanol), suggesting that the decision to continue forward or change direction after contact with an aversive stimulus is dictated largely by nutritional state. The 5-HT receptors modulating the initiation of reversal also modulate post-initiation behaviors, suggesting that nutritional status is defined by an extrasynaptic “serotonergic circuit.” The presence of food is perceived by a subset of sensory neurons and modulates many sensory-mediated behaviors. For example, the AWCs, ASKs, and ASIs are involved in locomotory changes associated with removal from food [6]. The AWCs are required for turning and reversals [7], the ASKs and AWCs for long reversals and omega turns, and the ASIs for dispersal during starvation [7], [16], [37]. Nutritional status also modulates odor conditioning in the AWCs and salt conditioning in the ASEs [47]–[50]. Interestingly, the ablation of sensory input has no apparent effect on reversal frequency on food, but dramatically alters reversal/turning off food, i.e., off food animals with compromised sensory input behave as if they are on food [6], [17]. Since, *tph-1* null animals on food behave as if they are off food, the “serotonergic circuit” identified in the present study may also mediate other food-dependent locomotory transitions.

The present study has demonstrated that food availability is translated by complex changes in both monoaminergic and peptidergic signaling to modulate aversive responses mediated by the ASHs and highlights the advantages of this model system for dissecting nutritional modulation, as these same excitatory and inhibitory food signals most certainly also modulate other nutritionally-dependent behaviors, including attraction, feeding, locomotion, and egg-laying. Given the advantages of the *C. elegans* model, it should now be possible to fully dissect these nutritionally-sensitive signaling pathways in the modulation of individual neurons or circuits.

## Supporting Information

Table S1 **Creation of rescue and overexpression transgenes.** Table includes all sense and antisense primers used for the generation of full-length rescue, neurons specific rescue or overexpression constructs, generated by PCR fusion [**25**].
(DOCX)

Table S2 **Creation of Neuron specific/selective RNAi knockdown transgenes.** Table represents all sense and antisense primers used for the generation of neuron specific/selective RNAi constructs, using PCR fusion [**25]**, [27****].
(DOCX)

Strain List S1 **The strain list represents all strains made and examined in present study.** The list includes all rescue, overexpressor and neuron specific/selective RNAi expressing animals that were generated for examination in octanol avoidance or post-initiation assays.
(DOCX)

## References

[1] Dacks AM, Christensen TA, Hildebrand JG (2008). Modulation of olfactory processing in the antennal lobe of *Manduca sexta* by serotonin.. J Neurophysiol.

[2] Petzold GC, Hagiwara A, Murthy VN (2009). Serotonergic modulation of odor input to the mammalian olfactory bulb.. Nature Neurosci.

[3] Savigner A, Duchamp-Viret P, Grosmaitre X, Chaput M, Garcia S (2009). Modulation of spontaneous and odorant-evoked activity of rat olfactory sensory neurons by two anorectic peptides, insulin and leptin.. J Neurophysiol.

[4] Trudeau LE (2004). Glutamate co-transmission as an emerging concept in monoamine neuron function.. J Psychiatry Neurosci.

[5] Hokfelt T, Broberger C, Xu ZQ, Sergeyev V, Ubink R (2000). Neuropeptides: an overview.. Neuropharmacology.

[6] Gray JM, Hill JJ, Bargmann CI (2005). A circuit for navigation in *Caenorhabditis elegans*.. Proc Natl Acad Sci USA.

[7] Chalasani SH, Chronis N, Tsunozaki M, Gray JM, Ramot D (2007). Dissecting a circuit for olfactory behaviour in *Caenorhabditis elegans*.. Nature.

[8] Chase DL, Koelle MR (2007). Biogenic amine neurotransmitters in *C. elegans*.. http://www.wormbook.org.

[9] Hapiak VM, Hobson RJ, Hughes LJ, Smith KA, Harris GP (2009). Dual excitatory and inhibitory serotonergic inputs modulate egg-laying in *Caenorhabditis elegans*.. Genetics.

[10] Chao MY, Komatsu H, Fukuto HS, Dionne HM, Hart AC (2004). Feeding status and serotonin rapidly and reversibly modulate a *Caenorhabditis elegans* chemosensory circuit.. Proc Natl Acad Sci USA.

[11] Harris GP, Hapiak VM, Wragg RT, Miller SB, Hughes LJ (2009). Three distinct amine receptors operating at different levels within the locomotory circuit are each essential for the serotonergic modulation of chemosensation in *Caenorhabditis elegans*.. J Neurosci.

[12] Harris G, Mills H, Wragg R, Hapiak V, Castelletto M (2010). The monoaminergic modulation of sensory-mediated aversive responses in *Caenorhabditis elegans* requires glutamatergic/peptidergic cotransmission.. J Neurosci.

[13] Sze JY, Victor M, Loer C, Shi Y, Ruvkun G (2000). Food and metabolic signaling defects in a *Caenorhabditis elegans* serotonin-synthesis mutant.. Nature.

[14] Chang AJ, Chronis N, Karow DS, Marletta MA, Bargmann CI (2006). A distributed chemosensory circuit for oxygen preference in *C. elegans*.. PLoS Biol.

[15] Zhang Y, Lu H, Bargmann CI (2005). Pathogenic bacteria induce aversive olfactory learning in *Caenorhabditis elegans*.. Nature.

[16] Wakabayashi T, Kitagawa I, Shingai R (2004). Neurons regulating the duration of forward movement in *Caenorhabditis elegans*.. Neurosci Res.

[17] Liang B, Moussaif M, Kuan CJ, Gargus JJ, Sze JY (2006). Serotonin targets the DAF-16/FOXO signaling pathway to modulate stress responses.. Cell Metab.

[18] Sawin ER, Ranganathan R, Horvitz HR (2000). *C. elegans* locomotory rate is modulated by the environment through a dopaminergic pathway and by experience through a serotonergic pathway.. Neuron.

[19] Brenner S (1974). The genetics of *Caenorhabditis elegans*.. Genetics.

[20] Sulston J, Hodgkin J, Wood WB, the Community of *C. elegans* Researchers (1988). Methods.. The Nematode *Caenorhabditis elegans*.

[21] Tsalik EL, Hobert O (2003). Functional mapping of neurons that control locomotory behavior in *Caenorhabditis elegans*.. J Neurobiol.

[22] Dernovici S, Starc T, Dent JA, Ribeiro P (2006). The serotonin receptor SER-1 (5HT2ce) contributes to the regulation of locomotion in *Caenorhabditis elegans*.. J Neurobiol.

[23] Pierce-Shimomura JT, Morse TM, Lockery SR (1999). The fundamental role of pirouettes in *C. elegans* chemotaxis.. J Neurosci.

[24] Mello C, Fire A (1995). DNA transformation.. Methods Cell Biol.

[25] Hobert O (2002). PCR fusion-based approach to create reporter gene constructs for expression analysis in transgenic *C. elegans*.. Biotechniques.

[26] Kramer JM, French RP, Park EC, Johnson JJ (1990). The *Caenorhabditis elegans rol-6* gene, which interacts with the *sqt-1* collagen gene to determine organismal morphology, encodes a collagen.. Mol Cell Biol.

[27] Esposito G, Di Schiavi E, Bergamasco C, Bazzicalupo P (2007). Efficient and cell specific knock-down of gene function in targeted *C. elegans* neurons.. Gene.

[28] Esposito G, Amoroso MR, Bergamasco C, Di Schiavi E, Bazzicalupo P (2010). The G protein regulators EGL-10 and EAT-16, the Giα GOA-1 and the G(q)α EGL-30 modulate the response of the C. elegans ASH polymodal nociceptive sensory neurons to repellents.. BMC Biol.

[29] Ezak MJ, Ferkey DM (2010). The *C. elegan*s D2-like dopamine receptor DOP-3 decreases behavioral sensitivity to the olfactory stimulus 1-octanol.. Plos One.

[30] Ezcurra MY, Tanizawa Y, Swoboda P, Schafer WR (2011). Food sensitizes *C. elegans* avoidance behaviors through acute dopamine signaling.. EMBO J.

[31] Sze JY, Zhang S, Li J, Ruvkun G (2002). The *C. elegans* POU-domain transcription factor UNC-86 regulates the tph-1 tryptophan hydroxylase gene and neurite outgrowth in specific serotonergic neurons.. Development.

[32] Ranganathan R, Sawin ER, Trent C, Horvitz HR (2001). Mutations in the *Caenorhabditis elegans* serotonin reuptake transporter MOD-5 reveal serotonin-dependent and –independent activities of fluoxetine.. J Neurosci.

[33] Nathoo AN, Moeller A, Westlund BA, Hart AC (2001). Identification of neuropeptide-like protein gene families in *Caenorhabditis elegans* and other species.. Proc Natl Acad Sci USA.

[34] Pierce SB, Costa M, Wisotzkey R, Devadhar S, Homburger SA (2001). Regulation of DAF-2 receptor signaling by human insulin and ins-1, a member of the unusually large and diverse *C. elegans* insulin gene family.. Genes Dev.

[35] Wu Q, Zhao Z, Shen P (2005). Regulation of aversion to noxious food by *Drosophila* neuropeptide Y- and insulin-like systems.. Nat Neurosci.

[36] Julliard AK, Chaput MA, Apelbaum A, Aime P, Mahfouz M (2007). Changes in rat olfactory detection performance induced by orexin and leptin mimicking fasting and satiation.. Behav Brain Res.

[37] Chalasani SH, Kato S, Albrecht DR, Nakagawa T, Abbott LF, Bargmann CI (2010). Neuropeptide feedback modifies odor-evoked dynamics in *Caenorhabditis elegans* olfactory neurons.. Nature Neurosci.

[38] Giachetti I, Mac Leod P, Le Magnen J (1970). Influence of hunger and satiety states on responses of the olfactory bulb in rats.. J Physiol (Paris).

[39] Prud'homme MJ, Lacroix MC, Badonnel K, Gougis S, Baly C (2009). Nutritional status modulates behavioural and olfactory bulb Fos responses to isoamyl acetate or food odour in rats: roles of orexins and leptin.. Neuroscience.

[40] Kindt KS, Tam T, Whiteman S, Schafer WR (2002). Serotonin promotes G(o)-dependent neuronal migration in *Caenorhabditis elegans*.. EMBO J.

[41] Albertson DG, Thomson JN (1976). The pharynx of *Caenorhabditis elegans*.. Philos Trans R Soc Lond B Biol Sci.

[42] White JG, Southgate E, Thomson JN, Brenner S (1986). The structure of the nervous system of the nematode *Caenorhabditis elegans*.. Philos Trans R Soc Lond B Biol Sci.

[43] Ren P, Lim CS, Johnsen R, Albert PS, Pilgrim D (1996). Control of *C. elegans* larval development by neuronal expression of a TGF-beta homolog.. Science.

[44] Schackwitz WS, Inoue T, Thomas JH (1996). Chemosensory neurons function in parallel to mediate a pheromone response in *C. elegans*.. Neuron.

[45] Ranganathan R, Cannon SC, Horvitz R (2000). MOD-1 is a serotonin-gated chloride channel that modulates locomotory behavior in *C. elegans*.. Nature.

[46] Wragg R, Hapiak V, Miller S, Harris G, Gray J (2007). Tyramine and octopamine independently inhibit serotonin-stimulated aversive behaviors in *Caenorhabditis elegans* through two novel amine receptors.. J Neurosci.

[47] Tomioka M, Adachi T, Suzuki H, Kunitomo H, Schafer WR (2006). The insulin/PI 3-kinase pathway regulates salt chemotaxis learning in *Caenorhabditis elegans*.. Neuron.

[48] Torayama I, Ishihara T, Katsura I (2007). *Caenorhabditis elegans* integrates the signals of butanone and food to enhance chemotaxis to butanone.. J Neurosci.

[49] Hukema RK, Rademakers S, Janssen G (2008). Gustatory plasticity in *C. elegans* involves integration of negative cues and NaCl taste mediated by serotonin, dopamine, and glutamate.. Learn Mem.

[50] Colbert HA, Bargmann CI (1995). Odorant-specific adaptation pathways generate olfactory plasticity in *C. elegans*.. Neuron.

[51] Aspöck G, Ruvkun G, Burglin TR (2003). The *Caenorhabditis elegans* ems class homeobox gene *ceh-2* is required for M3 pharynx motoneuron function.. Development.

[52] Sagasti A, Hobert O, Troemel ER, Ruvkun G, Bargmann CI (1999). Alternative olfactory neuron fates are specified by the LIM homeobox gene *lim-4*.. Genes Dev.

[53] Brockie PJ, Madsen DM, Zheng Y, Mellem J, Maricq AV (2001). Differential expression of glutamate receptor subunits in the nervous system of *Caenorhabditis elegans* and their regulation by the homeodomain protein UNC-42.. J Neurosci.

[54] Alkema MJ, Hunter-Ensor M, Ringstad N, Horvitz HR (2005). Tyramine Functions Independently of Octopamine in the *Caenorhabditis elegans* Nervous System.. Neuron.

