# Supplementary material for: Dissecting the Serotonergic Food Signal Stimulating Sensory-Mediated Aversive Behavior in C. elegans
Source: PLoS One. 2011 Jul 21;6(7):e21897. doi: 10.1371/journal.pone.0021897 (PMC3140990; doi:10.1371/journal.pone.0021897)
Supplement: Table S1 — Creation of rescue and overexpression transgenes. Table includes all sense and antisense primers used for the generation of full-length rescue, neurons specific rescue or overexpression constructs, generated by PCR fusion [25]. (DOCX) [file pone.0021897.s001.docx]

**SUPPLEMENTARY TABLE S1: Primers used for creation of rescue/overexpressor strains:**

| **ceh-2p:ins-1(+)** |  |
| --- | --- |
| CEH-2PF | 5’-TGGGTGTCACATTTTCGGTGG-3’ |
| CEH-2R:INS-1OVERLAP | 5’-CTTGACGAAACCAGTACATTTTCACTCCGAAT  ATTAGAAAAAATAAG-3’ |
| INS-1F | 5’- ATGTACTGGTTTCGTCAAGTTTACAG -3’ |
| INS-1R(INC 3’UTR) | 5’-CAAATTTAAAAAGAATGGATGCATCAGACG-3’ |
| CEH-2PF* | 5’-CATTTTCGGTGGTCACGCA-3’ |
| INS-1R* | 5’-GACGACTGTGAAGTTGAGA-3’ |
| **ceh-2p::ins-1::gfp** |  |
| CEH-2PROMF | 5’-TGGGTGTCACATTTTCGGTGG-3’ |
| CEH-2R::INS-1 | 5’-CTTGACGAAACCAGTACATTTTCACTCCGAAT  ATTAGAAAAAATAAG-3’ |
| INS-1F | 5’- ATGTACTGGTTTCGTCAAGTTTACAG -3’ |
| GFPF | 5’-AGCTTGCATGCCTGCAGGTCG-3’ |
| GFPR(INC 3’UTR) | 5’-AAGGGCCCGTACGGCCGACTA-3’ |
| INS-1R:GFP | 5’-CGACCTGCAGGCATGCAAGCTATTATCGTCCT  GATTGCAGCAG-3’ |
| CEH-2PROMF* | 5’-CATTTTCGGTGGTCACGCA-3’ |
| INS-1R* | 5’-TGATTGCAGCAGAATGTTTTGA-3’ |
| CEH-2PROMF* | 5’-tgagatttgcgtggattgcat-3’ |
| GFPR(INC 3’UTR)* | 5’-GGAAACAGTTATGTTTGGTATATTGG-3’ |
| **srh-142p::ins-1(+)** |  |
| SRH-142PROMF | 5’-GGTCGCGAGCTTTGATTTCCTT-3’ |
| SRH-142R::INS-1 | 5’-GTAAACTTGACGAAACCAGTACATATTGGCAA  AAAGAAAAAAGAGGTGCAAATA-3’ |
| INS-1F | 5’- ATGTACTGGTTTCGTCAAGTTTACAG -3’ |
| INS-1R (INC 3’UTR) | 5’-TTTAAAAAGAATGGATGCATCAGAC-3’ |
| SRH-142PROM* | 5’-CTCCAGCTTGAAGGGAAATTG-3’ |
| INS-1R* | 5’-CTGTGAAGTTGAGATCAGGTCAAAC-3’ |
| **ceh-2p::tph-1::gfp(+)** |  |
| CEH-2PROMF | 5’-TGGGTGTCACATTTTCGGTGG-3’ |
| CEH-2R::TPH-1 | 5’-CATCTGAAACAACGAATCCATTTTCACTCCGAA  TATTAGAAAAAATAAG-3’ |
| TPH-1F | 5’-ATGCCATCTGAAACAACGAATCCAT-3’ |
| TPH-1R:GFP | 5’-CGACCTGCAGGCATGCAAGCTCAGGATGTAGT  GGAGAGCTC-3’ |
| GFPF | 5’-AGCTTGCATGCCTGCAGGTCG-3’ |
| GFPR(INC3’UTR) | 5’-AAGGGCCCGTACGGCCGACTA-3’ |
| CEH-2PROMF* | 5’-CATTTTCGGTGGTCACGCA-3’ |
| TPH-1R* | 5’-GTTGATGTCTGAGCGGAGA-3’ |
| CEH-2PROMF* | 5’-CATTTTCGGTGGTCACGCA-3’ |
| GFPR(INC 3’UTR)* | 5’-GGAAACAGTTATGTTTGGTATATTGG-3’ |
| **Srh-142p::tph-1::gfp(+)** |  |
| SRH-142PF | 5’-GGTCGCGAGCTTTGATTTCCTT-3’ |
| SRH-142P:TPH-1 | 5’-CCATCTGAAACAACGAATCCATATTGGCAAAAAG  AAAAAAGAGGTGCAAATA-3’ |
| TPH-1F | 5’-ATGGATTCGTTGTTTCAGATGGCATCC-3’ |
| TPH-1R:GFP | 5’-CGACCTGCAGGCATGCAAGCTCAGGATGTAG  TGGAGAGCT-3’ |
| SRH-142P* | 5’-AATTgctccagcttgaagg-3’ |
| TPH-1R* | 5’-GCGAGCAGGTTGATGTCTG-3’ |
| GFPF | 5’-AGCTTGCATGCCTGCAGGTCG-3’ |
| GFPR | 5’-AAGGGCCCGTACGGCCGACTA-3’ |
| SRH-142PF* | 5’-ctccagcttgaagggaaattg-3’ |
| GFPR* | 5’-GGAAACAGTTATGTTTGGTATATTGG-3’ |
|  |  |
