# Supplementary material for: Dissecting the Serotonergic Food Signal Stimulating Sensory-Mediated Aversive Behavior in C. elegans
Source: PLoS One. 2011 Jul 21;6(7):e21897. doi: 10.1371/journal.pone.0021897 (PMC3140990; doi:10.1371/journal.pone.0021897)
Supplement: Table S2 — Creation of Neuron specific/selective RNAi knockdown transgenes. Table represents all sense and antisense primers used for the generation of neuron specific/selective RNAi constructs, using PCR fusion [25] , [27]. (DOCX) [file pone.0021897.s002.docx]

**SUPPLEMENTARY TABLE S2: Primers used for creation of cell specific/selective RNAi knockdown:**

| **NSM primers:** |  |
| --- | --- |
| **ceh-2p::tph-1RNAi** |  |
| CEH-2PF | 5’-TGGGTGTCACATTTTCGGTGG-3’ |
| CEH-2R:TPH-1PRS | 5’-CAGTACGAGTTGGTGTGAATTTCACTCCGA  ATATTAGAAAAAATAAG-3’ |
| CEH-2R:TPH-1PRA | 5’-ATATTTGTGCAAAAAGAGTGATTTTTCACT  CCGAATATTAGAAAAAATAAG-3’ |
| TPH-1TF | 5’- CTCTTCACACCAACTCGTACT -3’ |
| TPH-1TR | 5’-ACATAATCACTCTTTTTGCACAAATATCC-3’ |
| CEH-2PF* | 5’-CATTTTCGGTGGTCACGCA-3’ |
| TPH-1TR* | 5’-CTTTTTGCACAAATATCCAAATCATAAATAC-3’ |
| TPH-1TF* | 5’-CCAACTCGTACTGTTCGTCG -3’ |
| **ceh-2p::ins-1RNAi** |  |
| CEH-2PF | 5’-TGGGTGTCACATTTTCGGTGG-3’ |
| CEH-2R:INS-1PRS | 5’-ttgacgaaaccagtacatccgaatattagaa  aaaataagtaaactagc-3’ |
| CEH-2R:INS-1PRA | 5’-tgcaatcaggacgataatccgaatattagaa  aaaataagtaaactagc-3’ |
| INS-1TF | 5’-ATGTACTGGTTTCGTCAAGTTTACAG -3’ |
| INS-1TR | 5’-TTATCGTCCTGATTGCAGCAGAATG-3’ |
| CEH-2PF* | 5’-CATTTTCGGTGGTCACGCA-3’ |
| INS-1TR* | 5’-CTGATTGCAGCAGAATGTTTTGAG-3’ |
| INS-1TF* | 5’-TGGTTTCGTCAAGTTTACAGACC -3’ |
| **ceh-2p::unc-86RNAi** |  |
| CEH-2PF | 5’-TGGGTGTCACATTTTCGGTGG-3’ |
| CEH-2R:UNC-86PRS | 5’-TCCCGAAAAGTAGTTGCTATAGCCGAATATTA  GAAAAAATAAGTAAACTAGC-3’ |
| CEH-2R:UNC-86PRA | 5’-TAAAATTAGGAGTCACACAGCCGAATATTAGA  AAAAATAAGTAAACTAGC-3’ |
| UNC-86TF | 5’- CTATAGCAACTACTTTTCGGGAAT -3’ |
| UNC-86TR | 5’-CCTGTGTGACTCCTAATTTTATTCT-3’ |
| CEH-2PF* | 5’-CATTTTCGGTGGTCACGCA-3’ |
| UNC-86TR* | 5’-ATTCTTCTCTGCTTGAAATGCTCT-3’ |
| UNC-86TF* | 5’-AACTACTTTTCGGGAATCCACG -3’ |
| **ceh-2p::mod-5RNAi** |  |
| CEH-2PF | 5’-TGGGTGTCACATTTTCGGTGG-3’ |
| CEH-2R:MOD-5PRS | 5’-TGAACTTCAAGGACTTTGTATTTTCACTCCG  AATATTAGAAAAAATAAG-3’ |
| CEH-2R:MOD-5PRA | 5’-ATTTTAACAATAACTGCTATCGTTTCACTCC  GAATATTAGAAAAAATAAG-3’ |
| MOD-5TF | 5’- ATACAAAGTCCTTGAAGTTCAAAAAT -3’ |
| MOD-5TR | 5’-CGATAGCAGTTATTGTTAAAATC-3’ |
| CEH-2PF* | 5’-CATTTTCGGTGGTCACGCA-3’ |
| MOD-5TR* | 5’-AATCATTGTAACTGCTCAGCG-3’ |
| MOD-5TF* | 5’-CGAAGTTCAAAAATCAACAGGATTC -3’ |
|  |  |
|  |  |
| **ADF primers:** |  |
| **srh-142p::tph-1RNAi** |  |
| SRH-142PF | 5’-GGTCGCGAGCTTTGATTTCCTT-3’ |
| SRH-142R:TPH-1PRS | 5’-CAGTACGAGTTGGTGTGAAATTGGCAAAAA  GAAAAAAGAGGTGCAA-3’ |
| SRH-142R:TPH-1PRA | 5’-ATATTTGTGCAAAAAGAGTGATTATTGGCA  AAAAGAAAAAAGAGGTGCAA-3’ |
| TPH-1TF | 5’-CTCTTCACACCAACTCGTACT -3’ |
| TPH-1TR | 5’-ACATAATCACTCTTTTTGCACAAATATCC-3’ |
| SRH-142PF* | 5’-CTCCAGCTTGAAGGGAAATTG-3’ |
| TPH-1TR* | 5’-CTTTTTGCACAAATATCCAAATCATAAATAC-3’ |
| TPH-1TF* | 5’-CCAACTCGTACTGTTCGTCG-3’ |
| **srh-142p::ins-1RNAi** |  |
| SRH-142PF | 5’-GGTCGCGAGCTTTGATTTCCTT-3’ |
| SRH-142R:INS-1PRS | 5’-TCTGTAAACTTGACGAAACCAGTATTGGCAA  AAAGAAAAAAGAGGTGC-3’ |
| SRH-142R:INS-1PRA | 5’-TCAAAACATTCTGCTGCAATCAGGATTGGCA  AAAAGAAAAAAGAGGTGC-3’ |
| INS-1TF | 5’-ATGTACTGGTTTCGTCAAGTTTACAG-3’ |
| INS-1TR | 5’-TTATCGTCCTGATTGCAGCAGAATG-3’ |
| SRH-142PF* | 5’-CTCCAGCTTGAAGGGAAATTG-3’ |
| INS-1TR* | 5’-CTGATTGCAGCAGAATGTTTTGAG-3’ |
| INS-1TF* | 5’-TGGTTTCGTCAAGTTTACAGACC-3’ |
| **srh-142p::osm-9RNAi** |  |
| SRH-142PF | 5’-GGTCGCGAGCTTTGATTTCCTT-3’ |
| SRH-142R:OSM-9PRS | 5’-ATTCACAGGCGGTACGGATGGCAAAAAGAA  AAAAGAGGTGC-3 |
| SRH-142R:OSM-9PRA | 5’- TTCCTGGTTTCCTTCTCGCAAGGGCAAAAAG  AAAAAAGAGGTGC-3 |
| OSM-9TF | 5’-atccgtaccgcctgtgaattgtt-3’ |
| OSM-9TR | 5’-CTTGCGAGAAGGAAACCAG-3’ |
| SRH-142PF* | 5’-CTCCAGCTTGAAGGGAAATTG-3’ |
| OSM-9TR* | 5’-GGAAACCAGGAAGATCGC-3’ |
| OSM-9TF* | 5’-cgcctgtgaattgttaacaattttg-3’ |
| **srh-142p::mod-5RNAi** |  |
| SRH-142PF | 5’-GGTCGCGAGCTTTGATTTCCTT-3’ |
| SRH-142R:MOD-5PRS | 5’-TGAACTTCAAGGACTTTGTATattggcaaaa  agaaaaaagaggtgcaa-3’ |
| SRH-142R:MOD-5PRA | 5’-ATTTTAACAATAACTGCTATCGattggcaaa  aagaaaaaagaggtgcaa-3’ |
| MOD-5TF | 5’-ATACAAAGTCCTTGAAGTTCAAAAAT -3’ |
| MOD-5TR | 5’-CGATAGCAGTTATTGTTAAAATC-3’ |
| SRH-142PF* | 5’-CTCCAGCTTGAAGGGAAATTG-3’ |
| MOD-5TR* | 5’-AATCATTGTAACTGCTCAGCG-3’ |
| MOD-5TF* | 5’-CTTGAAGTTCAAAAATCAACAGGATTC-3’ |
|  |  |
| **egl-47p::mod-5RNAi** |  |
| egl-47pf | 5’-actttttctttggaaacgttgagt-3’ |
| egl-47r:mod-5PRS | 5’-TGAACTTCAAGGACTTTGTATCTGATAAGG  TTCATTCATTTTAAATACA-3’ |
| EGL-47P::MOD-5PRA | 5’-ATTTTAACAATAACTGCTATCGCTGATAA  GGTTCATTCATTTTAAATACA-3’ |
| MOD-5TGF | 5’-ATACAAAGTCCTTGAAGTTCAAAAAT-3’ |
| MOD-5TGR | 5’-CGATAGCAGTTATTGTTAAAATC-3’ |
| EGL-47PF* | 5’-AACGTTGAGTgtcttggag-3’ |
| MOD-5TGR* | 5’-AATCATTGTAACTGCTCAGCG-3’ |
| MOD-5TGF* | 5’-CTTGAAGTTCAAAAATCAACAGGATTC-3’ |
