# Supplementary material for: Dissecting the Serotonergic Food Signal Stimulating Sensory-Mediated Aversive Behavior in C. elegans
Source: PLoS One. 2011 Jul 21;6(7):e21897. doi: 10.1371/journal.pone.0021897 (PMC3140990; doi:10.1371/journal.pone.0021897)
Supplement: Strain List S1 — The strain list represents all strains made and examined in present study. The list includes all rescue, overexpressor and neuron specific/selective RNAi expressing animals that were generated for examination in octanol avoidance or post-initiation assays. (DOCX) [file pone.0021897.s003.docx]

**Supplementary Material file:**

Dissecting the serotonergic food signal stimulating sensory-mediated aversive behavior in *C. elegans*

Gareth P Harris, Amanda C Korchnak, Philip J Summers, Vera M Hapiak, Wen Jing Law, Andrew M Stein, Patricia Komuniecki, and Richard W Komuniecki*

**RESCUE/Overexpressor strains:**

*RWK108 ins-1(tm1888); fvEx47[ceh-2p::ins-1::gfp*] expressed *ins-1* in the NSM neurons of *ins-1* null animals.

*RWK109 N2; fvEx47[ceh-2p::ins-1XS::gfp*] expressed *ins-1* in the NSM neurons of wild-type animals.

*RWK111 N2; fvEx49[ceh-2p::tph-1::gfpXS*] expressed *tph-1* in the NSM neurons of wild-type animals.

*RWK114 N2; fvEx51[srh-142p::ins-1XS*] expressed *ins-1* in the ADF sensory neurons of wild-type animals.

*RWK228 N2; fvEx108[srh-142p::tph-1XS::gfp]* expressed *tph-1* in the ADF sensory neurons of wild-type animals

**Neuron selective/specific RNAi strains:**

*RWK13 N2;* *fvEx6[glr-3p::ser-1RNAi]* expressed a ser-1 RNAi in the RIA interneurons of wild-type animals

*RWK20 N2; fvEx109[ceh-2p::tph-1RNAi]* expressed a tph-1 RNAi in the NSM neurons of wild-type animals

*RWK21 N2; fvEx8[srh-142p::tph-1RNAi]* expressed a tph-1 RNAi in the ADF sensory neurons of wild-type animals

*RWK124 N2; fvEx61[ceh-2p::ins-1RNAi]* expressed an ins-1 RNAi in the NSM neurons of wild-type animals

*RWK125 ser-1; fvEx61[ceh-2p::ins-1RNAi]* expressed an ins-1 RNAi in the NSM neurons of *ser-1(ok345)* animals

*RWK126 ser-5; fvEx61[ceh-2p::ins-1RNAi]* expressed an ins-1 RNAi in the NSM neurons of *ser-5(tm2654)* animals

*RWK127 mod-1; fvEx61[ceh-2p::ins-1RNAi]* expressed an ins-1 RNAi in the NSM neurons of *mod-1(ok103)* animals

*RWK128 ser-1; fvEx62[ceh-2p::mod-5RNAi]* expressed a mod-5 RNAi in the NSM neurons of *ser-1(ok345)* animals

*RWK128 N2; fvEx62[ceh-2p::mod-5RNAi]* expressed a mod-5 RNAi in the NSM neurons of wild-type animals

*RWK130 ser-5; fvEx62[ceh-2p::mod-5RNAi]* expressed a mod-5 RNAi in the NSM neurons of *ser-5(tm2654)* animals

*RWK131 mod-1; fvEx62[ceh-2p::mod-5RNAi]* expressed a mod-5 RNAi in the NSM neurons of *mod-1(ok103)* animals

*RWK132 N2; fvEX61/fvEX7[ceh-2p::ins-1/tph-1RNAi]* expressed a ins-1 and tph-1 RNAi in the NSM neurons of wild-type animals

*RWK134 N2; fvEx64[srh-142p::osm-9RNAi]* expressed an osm-9 RNAi in the ADF sensory neurons of wild-type animals

*RWK137 N2; fvEx67[srh-142p::ins-1RNAi]* expressed an ins-1 RNAi in the ADF sensory neurons of wild-type animals

*RWK138 ser-1; fvEx67[srh-142p::ins-1RNAi]* expressed an ins-1 RNAi in the ADF sensory neurons of *ser-1(ok345)* animals

*RWK139 ser-5; fvEx67[srh-142p::ins-1RNAi]* expressed an ins-1 RNAi in the ADF sensory neurons of *ser-5(tm2654)* animals

*RWK140 mod-1; fvEx67[srh-142p::ins-1RNAi]* expressed an ins-1 RNAi in the ADF sensory neurons of *mod-1(ok103)* animals

*RWK141 N2; fvEx68[srh-142p::mod-5RNAi]* expressed a mod-5 RNAi in the ADF sensory neurons of wild-type animals

*RWK142 ser-1; fvEx68[srh-142p::mod-5RNAi]* expressed a mod-5 RNAi in the ADF sensory neurons of *ser-1(ok345)* animals

*RWK143 ser-5; fvEx68[srh-142p::mod-5RNAi]* expressed a mod-5 RNAi in the ADF sensory neurons of *ser-5(tm2654)* animals

*RWK144 mod-1; fvEx68[srh-142p::mod-5RNAi]* expressed a mod-5 RNAi in the ADF sensory neurons of *mod-1(ok103)* animals

*RWK145 N2; fvEx8/fvEx67[srh-142p::tph-1/ins-1RNAi]* expressed a tph-1 and ins-1 RNAi in the ADF sensory neurons of wild-type animals

*RWK218 N2; fvEx68/fvEx6[srh-142p::mod-5/glr-3p::ser-1RNAi]* expressed a mod-5 and ser-1 RNAi in the ADFs and RIAs of wild-type animals

*RWK219 N2; fvEx67/fvEx6[srh-142p::ins-1/glr-3p::ser-1RNAi]* expressed an ins-1 and ser-1 RNAi in the ADFs and RIAs of wild-type animals

*RWK220 N; fvEx62/fvEx6[ceh-2p::mod-5/glr-3p::ser-1RNAi]* expressed a mod-5 and ser-1 RNAi in the NSMs and RIAs of wild-type animals

*RWK221 N2; fvEx61/fvEx6[ceh-2p::ins-1/glr-3p::ser-1RNAi]* expressed an ins-1 RNAi and ser-1 RNAi in the NSMs and RIAs of wild-type animals

*RWK224 N2; fvEx107[egl-47p::mod-5RNAi]* expressed a mod-5 RNAi in the HSNs of wild-type animals

*RWK225 mod-1; fvEx107[egl-47p::mod-5RNAi]* expressed a mod-5 RNAi in the HSNs of *mod-1(ok103)* animals

*RWK226 tdc-1; fvEx68[srh-142p::mod-5RNAi]* expressed a mod-5 RNAi in the ADFs of *tdc-1(n3419)* animals

*RWK227 tbh-1; fvEx68[srh-142p::mod-5RNAi]* expressed a mod-5 RNAi in the ADFs of *tbh-1(n3247)* animals

*RWK228 N2; fvEx[srh-142p::mod-5RNAi/glr-3p::ser-1RNAi]* expressed a mod-5 and ser-1 RNAi in the ADFs and RIAs of wild-type animals

*RWK229 N2; fvEx[srh-142p::ins-1RNAi/glr-3p::ser-1RNAi]* expressed an ins-1 and ser-1 RNAi in the ADFs and RIAs of wild-type animals
